# Multi-Omics Analysis of Anlotinib in Pancreatic Cancer and Development of an Anlotinib-Related Prognostic Signature

**DOI:** 10.3389/fcell.2021.649265

**Published:** 2021-03-04

**Authors:** Xi Zhang, Yang Liu, Zhen Zhang, Juan Tan, Junjun Zhang, Hao Ou, Jie Li, Zewen Song

**Affiliations:** ^1^Department of Oncology, The Third Xiangya Hospital of Central South University, Changsha, China; ^2^Department of Pathology, The Third Xiangya Hospital of Central South University, Changsha, China; ^3^Department of Information Science and Engineering, Hunan University of Chinese Medicine, Changsha, China

**Keywords:** anlotinib, pancreatic cancer, transcriptomics, proteomics, phosphoproteomics, ingenuity pathway analysis

## Abstract

Aberrant regulation of angiogenesis involves in the growth and metastasis of tumors, but angiogenesis inhibitors fail to improve overall survival of pancreatic cancer patients in previous phase III clinical trials. A comprehensive knowledge of the mechanism of angiogenesis inhibitors against pancreatic cancer is helpful for clinical purpose and for the selection of patients who might benefit from the inhibitors. In this work, multi-omics analyses (transcriptomics, proteomics, and phosphoproteomics profiling) were carried out to delineate the mechanism of anlotinib, a novel angiogenesis inhibitor, against pancreatic cancer cells. The results showed that anlotinib exerted noteworthy cytotoxicity on pancreatic cancer cells. Multi-omics analyses revealed that anlotinib had a profound inhibitory effect on ribosome, and regulated cell cycle, RNA metabolism and lysosome. Based on the multi-omics results and available data deposited in public databases, an anlotinib-related gene signature was further constructed to identify a subgroup of pancreatic cancer patients who had a dismal prognosis and might be responsive to anlotinib.

## Introduction

Aberrant regulation of angiogenesis facilitates the growth and metastasis of tumors (Folkman, 2007). Dozens of anti-angiogenic agents, through mechanisms by neutralizing vascular endothelial growth factors (VEGFs), targeting VEGF receptors (VEGFRs), or inhibiting multiple pro-angiogenic pathways, are effective in several types of cancer; and some of these agents have been approved for use in oncology (Ferrara et al., 2004; Han B. et al., 2018; Kudo et al., 2018; Qin et al., 2019). However, evidence from clinical trials suggested that not all types of cancer respond well to this strategy, although angiogenesis is regarded as a shared mechanism in the progress of tumors (Kindler et al., 2010; Ohtsu et al., 2011; Cheng et al., 2013). For instance, all phase III clinical trials, to our knowledge, fail to show that angiogenesis inhibitors, either alone or combining with chemotherapy, significantly improve overall survival (OS) of pancreatic cancer patients, leading to a universal agreement that targeting VEGF signaling is an ineffective strategy in this disease (Korc, 2003; Kindler et al., 2010, 2011; Rougier et al., 2013; Yamaue et al., 2015). Some explanations have been proposed, including induced tumor hypoxia triggering VEGF production and vascular permeability, negative effects on the delivery of anti-tumor drugs, and tumor vascularization via alternative mechanisms (Annese et al., 2019).

Cancer patients have distinct sensitivity to anti-cancer treatments. This sensitivity, supported by numerous studies, can be modulated by aberrant expression of some genes. For instance, overexpression of some ATP-binding cassette (ABC) transporters in tumors, such as ABCB1, is frequently associated with insensitivity to anti-cancer drugs by decreasing cellular accumulation of the drugs (Huang and Sadee, 2006). Inhibition of DNA damage inducible transcript 4 (DDIT4) in bladder urothelial carcinoma renders the cancer cells more sensitive to paclitaxel by inhibiting autophagy (Zeng et al., 2018). In recent years, researchers are trying to identify subgroups of tumor patients, based on their molecular profile, that have distinct treatment response and different prognosis (Jackson and Chester, 2015; Abubakar and Gan, 2016; Jia et al., 2016; Zhou Z. et al., 2019). Although phase III clinical trials do not prove that angiogenesis inhibitors can improve the OS of pancreatic cancer patients; some of them, with specific molecular profile, might obtain survival benefit from these agents. After all, *in vitro* experiments, animal studies and some phase I/II clinical studies indicate that anti-angiogenic therapy is effective in pancreatic cancer (Korc, 2003; Kindler et al., 2010, 2011; Rougier et al., 2013; Yamaue et al., 2015; Zhang et al., 2018).

Anlotinib is a novel multi-tyrosine kinase inhibitor (TKI) and its anti-angiogenic activity seems stronger than that of other anti-angiogenesis drugs (Lin et al., 2018; Shen et al., 2018; Xie et al., 2018). ALTER 0303 study showed that anlotinib as third line treatment substantially prolongs the OS of advanced non-small cell lung cancer (NSCLC) patients than those received placebo treatment (9.6 months vs. 6.3 months, *p* = 0.002) (Han B. et al., 2018). Other clinical evidence suggested that the inhibitor is also effective in treating soft-tissue sarcoma (STS) and medullary thyroid carcinoma (MTC) (Chi et al., 2018; Sun et al., 2018). Recently, the agent has been approved as a third-line treatment for NSCLC and SCLC, and as a first-line or second-line treatment for some subtypes of STS in China.

In this study, we intended to get a comprehensive knowledge of anlotinib against pancreatic cancer by conducting multi-omics (transcriptomics, proteomics and phosphoproteomics) analyses. The results showed that anlotinib was cytotoxic to pancreatic cancer cells. The inhibitor had a remarkable inhibitory effect on ribosome, and regulated cell cycle, RNA metabolism and lysosome. Based on the multi-omics profiling and available data deposited in public databases like the Cancer Genome Atlas (TCGA), we further constructed an anlotinib-related gene signature, which identified a subgroup of pancreatic cancer patients who had a dismal prognosis and might be responsive to the drug.

## Materials and Methods

### Reagent

Anlotinib was kindly provided by the CTTQ Pharma (Lianyungang, China). The compound was dissolved in dimethylsulfoxide (DMSO) to 10 mM as stock solution and stored at −20°C, as reported in a previous study (Lin et al., 2018). The stock solution was then diluted with medium before each experiment.

### Cell Culture

AsPC-1 cells were obtained from the cell bank of Chinese Academy of Sciences Cell Bank (Shanghai, China) while PANC-1 cells were from American Type Culture Collection (ATCC, United States). Both cell lines were confirmed to be free of mycoplasma before experiments. Cells were cultured in RPMI-1640 medium (Invitrogen, United States) supplemented with 10% fetal bovine serum (FBS, ExCell), and were incubated under humidified atmospheric conditions with 5% CO_2_ at 37°C.

### CCK-8 Assay

Cell Counting Kit-8 (CCK-8) assay was used to evaluate cell proliferation. Briefly, PANC-1 and AsPC-1 cells were seeded at a density of 4000 cells per well in 96-well plates and incubated for 1, 2, 3, 4, or 5 days respectively. Ten μl CCK-8 (Dojindo Molecular Technologies, Japan) was added to each well, incubated for 4 h, and mixed gently on an orbital shaker for 2 min before absorbance value (OD) of each well was measured at 450 nm. Experiments were carried out in triplicate.

### Cell Cycle and Apoptosis Assay

Cells were seeded on 6 cm-diameter plates with RPMI-1640 containing 10% FBS. After treatment, cells were labeled by using a cell-cycle detection Kit (Sigma, United States) and annexin V-FITC/PI staining kit (eBioscience, United States), according to manufacturer’s instructions. The DNA content of labeled cells was analyzed with FACS cytometry (Millipore, United States). Experiments were performed in triplicate.

### Cell Invasion Assay

1 × 10^5^ transfected cells were seeded in 500 μl RPMI-1640 medium on the matrigel in the upper chamber of the Corning^®^ BioCoat^TM^ Matrigel^®^ Invasion Chambers (8 mm pore size; Corning, United States), 750 μl RPMI-1640 medium containing 30% FBS was added in the bottom chamber. The cells were incubated for 24 h at 37°C with 5% CO_2_ and then were fixed in 4% paraformaldehyde and stained with Giemsa Stain solution (Sigma, United States). The cells on the bottom of the membrane were visualized under a microscope and quantified by counting the number of cells in three randomly chosen fields at 100-fold magnification. Experiments were performed in triplicate.

### Wound Healing Assay

Pancreatic cancer cells were seeded in 96 well plates at a density of 5 × 10^4^ per well and cultured overnight. When the cells reached to the 90% confluency, wounds were created with a 96 Wounding Replicator (VP Scientific, United States). The cells were washed gently with culture medium and further cultured with medium containing 1% FBS. Images were acquired at three different time points, namely 0, 8, and 24 h with a Celigo Image Cytometer (Nexcelom Bioscience, United States) at 100x magnification. The wound healing rate was also quantified by the Celigo Image Cytometer. Experiments were performed in triplicate.

### RNA Extraction and qRT-PCR

Total RNA was isolated using Trizol (Pufei Biotech Co., Ltd, Shanghai, China) and then quantified with the Nanodrop 1000 (Thermo Fisher Scientific, United States). An M-MLV reverse transcription reagent kit (Promega, United States) was used to perform reverse transcription according to the protocol. Quantitative PCR analysis was performed with LightCycler 480 II (Roche, Germany) using SYBR Master Mixture (TAKARA, Japan). The primers used were constructed by Genechem Technologies (Shanghai, China) and were included in Supplementary Table 1. Expression data of all genes were normalized against the internal control gene GAPDH.

### Transcriptome Profiling

Panc-1 cells were treated with anlotinib or DMSO as control before RNA extraction. Three samples from each group were used for experiments. RNA quality and integrity was measured by Thermo Nanodrop 2000 (Thermo Fisher Scientific, United States) and Agilent 2100 Bioanalyzer (Agilent, United States). Poly-A RNA control addition, cDNA systhesis, labeling of cRNA, cRNA purification, fragmentation, and hybridization were performed by Genechem Technologies (Shanghai, China), using GeneChip^TM^ 3′ IVT PLUS reagent kit, and GeneChip^TM^ hybridization, wash, and stain Kit (Thermo Fisher Scientific, United States). Data were extracted by using GeneChip^TM^ Scanner 3000 (Thermo Fisher Scientific, United States). R (version 3.6.2), and the following packages: bioconductor, limma, affy, lattice, pcaGoPromoter, graphics, and gplots, were used for background adjustment, normalization, quality control, and statistical analysis of differentially expressed genes (DEGs). Probe sets were filtered if their signal intensity were in the lowest 20% arrange or their intra-class coefficient of variation (CV) was greater than 25%.

Differential gene expression levels between the two groups were estimated with moderated *t*-test via the limma package in R software. Benjamini–Hochberg algorithm (false discovery rate, FDR) was applied for the correction of *p*-values. DEGs were defined with the following screening criteria: absolute fold-change ≥ 2 and FDR < 0.05. DEGs (gene identifiers) and their corresponding expression values were uploaded into the ingenuity pathway analysis (IPA) software (Qiagen, Germany). Each gene identifier was plotted to its similar gene entity in the Ingenuity Pathways Knowledge Base (IPKB). The “core analysis” function of the software was applied for the interpretation of the differentially expressed data, which included canonical pathways, upstream regulators, diseases and biofunction, and regulator effect networks.

The transcriptomics data had been deposited in the GEO database with the accession number GSE163574.

### Proteomics and Phosphoproteomics Data Acquisition

Pancreatic cancer cells treated with anlotinib or DMSO (three biological replicates for each condition) for 48 h were added with SDT buffer (4% SDS, 100 mM DTT, 150 mM Tris-HCl pH 8.0). For each sample, the lysate was sonicated and then boiled for 15 min. The supernatant of each sample was separated after centrifugation at 14000 *g* for 40 min, and was quantified with the BCA Protein Assay Kit (P0012, Beyotime). Two hundred μg proteins for each sample were performed protein digestion according to the filter-aided sample preparation (FASP) procedure described by Wisniewski et al. (2009). Briefly, proteins of each sample were added into 30 μl SDT buffer. The mixture was put it in a boiled water bath for 5 min. UA buffer (8 M Urea, 150 mM Tris-HCl pH 8.5) was added to remove the detergent, DTT and other low-molecular-weight components by repeated ultrafiltration (Sartorius, 30 kD). Reduced cysteine residues were blocked by adding 100 μl iodoacetamide (100 mM IAA in UA buffer) and the samples were incubated without light for 30 min. Hundred μl UA buffer and then 100 μl 0.1M TEAB buffer were used to wash the filters. At last, 40 μL trypsin buffer (4 μg trypsin in 40 μl 0.1M TEAB buffer) was used to digest protein suspensions for 18 h at 37°C. The generated peptides were collected as a filtrate. Hundred μg peptide mixture of each sample was then labeled using Tandem Mass Tags^TM^ (TMT) reagent according to the manufacturer’s instructions (Thermo Fisher Scientific, United States).

For Proteomic analysis, TMT labeled peptides were fractionated by RP chromatography using the Agilent 1260 infinity II HPLC. Buffer A (10 mM HCOONH4, 5% ACN, pH 10.0) and B (10 mM HCOONH4, 85% ACN, pH 10.0) were used for fractionation. The peptide mixture was diluted with buffer A and loaded onto a XBridge Peptide BEH C18 Column, 130 Å, 5 μm, 4.6 mm × 100 mm column (Waters, United States). The peptides were eluted at a flow rate of 1 ml/min with a gradient of 0% buffer B for 25 min, 0–7% buffer B during 25 – 30 min, 7 – 40% buffer B during 30 – 65 min, 40 – 100% buffer B during 65 – 70 min, 100% buffer B during 70 – 85 min. The elution was monitored at 214 nm based on the UV light trace, and fractions were collected every 1 min. Approximate 40 fractions were collected and dried down via vacuum centrifugation at 45°C. The fractions were then dissolved with 0.1% FA and combined into 10 fractions.

For phosphoproteomic analysis, the labeled peptides were combined and desalted using C18 Cartridge. The peptides mixture was subjected to HiSelect TiO2 phosphopeptide enrichment kit (Thermo Fisher Scientific, United States). The TiO2 flow-through (FT) and wash fractions were pooled, and the phosphopeptides were enriched by HiSelect Fe-NTA phosphopeptide enrichment kit (Thermo Fisher Scientific, United States). The TiO2 eluent and Fe-NTA eluent were dried down via vacuum centrifugation at 45°C and then dissolved in 0.1% Formic acid buffer.

LC-MS/MS analysis was conducted on a Q Exactive HF-X mass spectrometer (Thermo Fisher Scientific, United States) that was coupled to Easy nLC (Thermo Fisher Scientific, United States). Each eluent was injected for nanoLC-MS/MS analysis twice. The peptide mixture was loaded onto the C18-reversed phase analytical column (Thermo Fisher Scientific, United States) in buffer A (0.1% Formic acid) and separated with a linear gradient of buffer B (80% acetonitrile and 0.1% Formic acid) at a flow rate of 300 nl/min.

The mass spectrometer was operated in positive ion mode. MS data was acquired using a data-dependent top 10 method dynamically choosing the most abundant precursor ions from the survey scan (350–1800 m/z) for HCD fragmentation. Survey scans were acquired at a resolution of 60000 at m/z 200 with an AGC target of 3e6 and a maxIT of 50 ms. MS2 scans were acquired at a resolution of 15000 for HCD spectra at m/z 200 with an AGC target of 2e5 and a maxIT of 120 ms, and isolation width was 2 m/z. Only ions with a charge state between 2 and 6 and a minimum intensity of 2e3 were selected for fragmentation. Dynamic exclusion for selected ions was 30 s. Normalized collision energy was 30 eV.

MS/MS raw files were processed using MASCOT engine (Matrix Science, London, United Kingdom; version 2.6) embedded into Proteome Discoverer 2.2 (Thermo Fisher Scientific, United States), a software used for quantification of proteins. The Uniprot Human database was searched and downloaded on February 26th, 2020, including 71,090 sequences. The search parameters included trypsin as the enzyme used to generate peptides with a maximum of two missed cleavages permitted. A precursor mass tolerance of 10 ppm was specified and 0.05 Da tolerance for MS2 fragments. Except for TMT labels, carbamidomethyl (C) was set as a fixed modification. Variable modifications were Oxidation (M), Acetyl (Protein N-term), Phospho (ST), and Phospho (Y). A peptide and protein false discovery rate of 1% was enforced using a reverse database search strategy (Elias and Gygi, 2007). Proteins or phosphopeptides with Fold change > 1.2 and *p*-value (Student’s *t*-test) < 0.05 were considered to be differentially expressed proteins or phophopeptides.

The proteomics and phosphoproteomics data had been submitted to the ProteomeXchange database with the accession numbers PXD023344 and PXD023345.

### Bioinformatics Analysis

Acquisition and processing of pancreatic cancer related public data sets from the Cancer Genome Atlas (TCGA_PAAD), the International Cancer Genome Consortium (ICGC_AU) and the Gene Expression Omnibus (GEO, GSE62452) were conducted as reported in our previous study (Zhang et al., 2020). The series matrix file of the GSE62452 data set was downloaded via the GEOquery package in the R software. After data filtering, 176 tumor samples with survival data in TCGA_PAAD, 80 tumor samples with survival data in ICGC_AU, and 65 samples in GSE62452 were used for further analysis.

Gene ontology (GO), Kyoto Encyclopedia of Genes and Genomes (KEGG) enrichment analysis and gene set enrichment analysis (GSEA) were conducted as reported in our previous studies (Yu et al., 2012; Qu et al., 2020; Zhang et al., 2020).

For the construction of the anlotinib-related signature, genes of interest were underwent the least absolute shrinkage and selection operator (LASSO) Cox regression analysis via the glmnet package in R. The LASSO Cox analysis generated five crucial genes, which were further underwent multivariate Cox regression analysis to generate the corresponding coefficient. A new score was calculated by multiplying the normalized gene expression of each gene and its corresponding coefficient, and the formula was score = 0.15289 ^∗^ TOP2A + 0.07895 ^∗^ CRABP2 + 0.01827 ^∗^ CDK1 + 0.31132 ^∗^ NUSAP1 + 0.24815 ^∗^ PERP. To facilitate the interpretation of results across different data sets, the risk score was calculated with the formula reported in our previous study, namely, risk score = (score-Min)/absolute(Max) (Qu et al., 2020).

### Statistical Analysis

For *in vitro* experiments, data were presented as the mean ± SD. Student’s *t*-test was used to determine statistical significance between two groups. Data were graphically displayed using GraphPad Prism v.8.0.1 for Windows (GraphPad Software, Inc., La Jolla, CA, United States).

Patients were divided into two subgroups based on the optimal cut-off value of the risk score via the surv_cutpoint function of the survminer package (R software, version 3.6). Univariate and multivariate Cox regression, survival analyses and time-dependent receiver operator characteristic (ROC) analyses were conducted as reported in our previous study (Qu et al., 2020). Significance of difference was indicated as ^∗^*P* < 0.05; ^∗∗^*P* < 0.01; ^∗∗∗^*P* < 0.001; ^****^P < 0.0001.

## Results

### Anlotinib Was Cytotoxic to Pancreatic Cancer Cells

**FIGURE 1:**
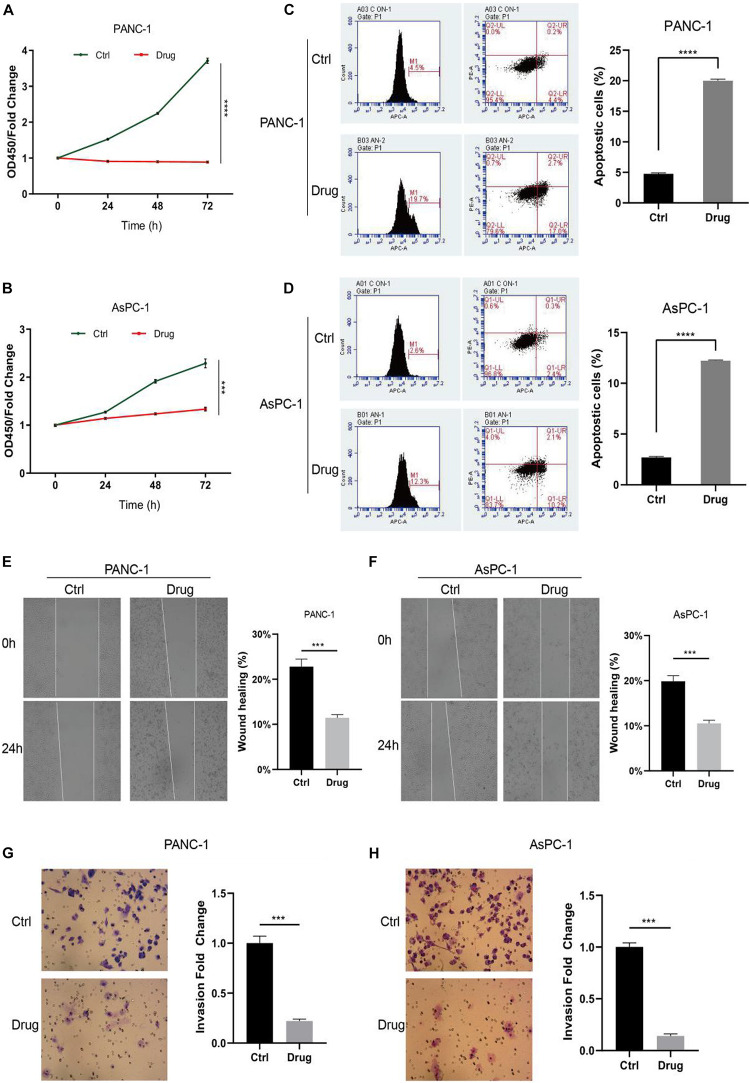
Anlotinib was cytotoxic to pancreatic cancer cells. **(A,B)** CCK-8 assay of PANC-1 **(A)** or AsPC-1 **(B)** cells treated with anlotinib or DMSO. **(C,D)** Apoptosis assay of PANC-1 **(C)** or AsPC-1 **(D)** cells treated with anlotinib or DMSO. **(E,F)** Wound healing assay of PANC-1 **(E)** or AsPC-1 **(F)** cells treated with anlotinib or DMSO. **(G,H)** Invasion assay of PANC-1 **(G)** or AsPC-1 **(H)** cells treated with anlotinib or DMSO. Data were shown in mean ± SD and *p* < 0.05 was statistically significant. ***p < 0.001; ****p < 0.0001.

To investigate the impact of anlotinib on pancreatic cancer cells, we first calculated its IC50 values in two cancer cell lines, namely AsPC-1 and PANC-1. Cells were treated with anlotinib at increasing concentration for 48 h. A dose-dependent growth inhibition of anlotinib, from 0 to 25 μM, was observed on both pancreatic cancer cell lines, and the IC50 values were calculated to be 5.535 and 4.642 μM for PANC-1 and AsPC-1 cells, respectively (Supplementary Figures 1A,B). When these cells was treated with the drug at the corresponding IC50 concentration, both pancreatic cell lines showed a dramatically decline in cell proliferation on day 5 (Figures 1A,B, *p* < 0.001). A more than threefold increase in the ratio of apoptotic cells occurred in pancreatic cancer cells treated with anlotinib for 48 h (Figures 1C,D, *p* < 0.0001). The wound healing assay showed that cancer cells treated with the drug had a significantly decline in the migration capability (Figures 1E,F, *p* < 0.001). In addition, the number of pancreatic cancer cells treated with anlotinib was approximately 20% of that of the control in invading through the matrigel (Figures 1G,H, *p* < 0.001). Taken together, these data indicated that anlotinib exerted considerable cytotoxicity on pancreatic cancer cells.

### Transcriptomics Analysis

**FIGURE 2:**
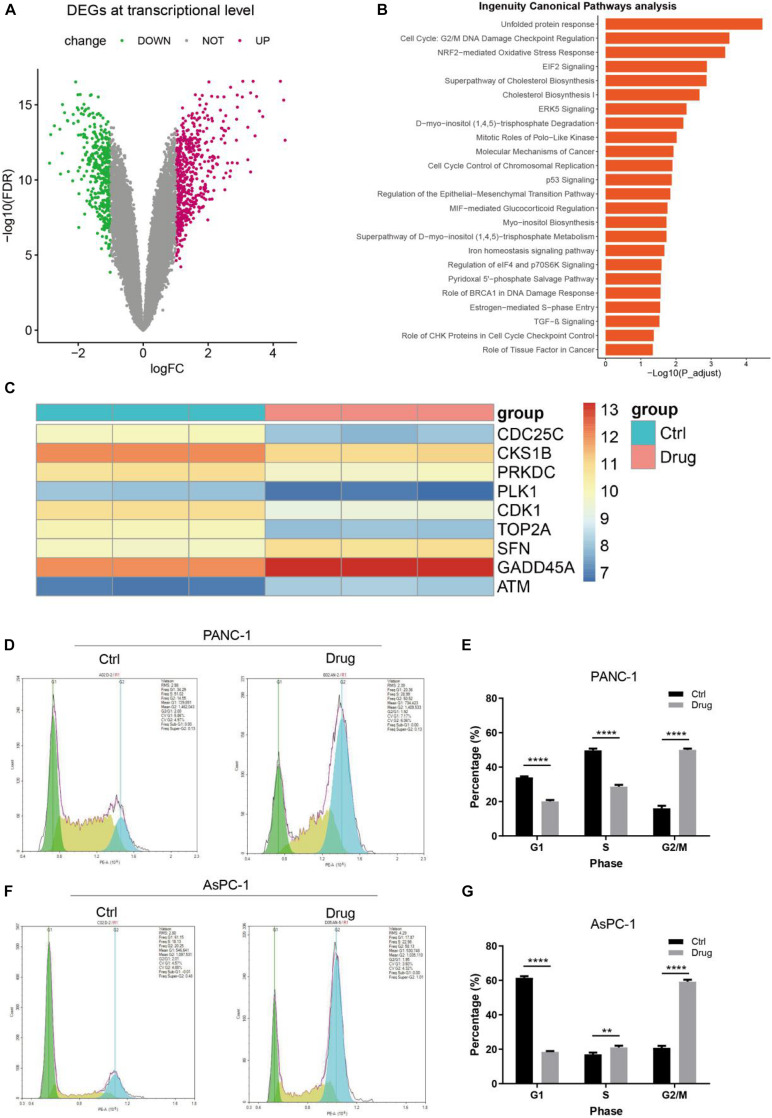
Transcription profiling and canonical pathway analysis. **(A)** The volcano plot of differentially expressed genes (DEGs) of PANC-1 cells treated with anlotinib. **(B)** Canonical pathways analysis of the DEGs via the IPA software. **(C)** The heatmap of the DEGs related to *cell cycle: G2/M DNA damage checkpoint regulation*. **(D,E)** Cell cycle assay of PANC-1 cells treated with anlotinib or DMSO. **(F,G)** Cell cycle assay of AsPC-1 cells treated with anlotinib or DMSO. Data were shown in mean ± SD and *p* < 0.05 was statistically significant. **p < 0.01; ****p < 0.0001.

To shed light on the underlying mechanism of anlotinib in suppressing pancreatic cancer cells, we first conducted transcriptomics analysis. PANC-1 cells were treated with anlotinib or DMSO as control for 48 h and extracted their total RNA for transcriptome profiling. The generated raw data was first normalized and underwent quality control (QC) evaluation (Supplementary Figures 1C–E), which suggested that the six samples were of sufficiently high quality for further analysis. After data filtering, 39,219 probe sets were included in the differential expression analysis. Overall, 992 genes were significantly deferentially expressed after data processing, with the following screening criteria: fold change ≥ 2 and FDR < 0.05 (Figure 2A). RT-PCR analysis of 30 randomly selected genes (15 up-regulated and 15 down-regulated respectively) showed that the regulation of these genes were consistent with the transcriptome profiling analysis (Supplementary Figures 1F,G and Supplementary Table 2). Then we were interested to see which signaling pathways were affected by anlotinib. These DEGs and their corresponding expression values were uploaded into the IPA software (Qiagen) for canonical pathways analysis. As shown in Figure 2B, 24 cancer-related canonical pathways were significantly affected [–log (*p*-value) > 1.301]. Unfolded protein response was the most significantly affected pathways (Figure 2C), suggesting a possible ER stress caused by the drug. In addition, several affected pathways were associated with DNA damage and cell cycle regulation, like *Cell cycle: G2/M DNA damage checkpoint regulation*, *cell cycle control of chromosomal replication*, and *role of BRCA1 in DNA damage response* (Figure 2B). ATM serine/threonine kinase (ATM) (Li et al., 2016), stratifin (SFN) (Fomenkov et al., 2004), and growth arrest and DNA damage 45 alpha (GADD45A) (Wingert et al., 2016), which would be activated and arrest cell cycle in response to DNA damage, were up-regulated in pancreatic cancer cells after anlotinib treatment (Figure 2C and Supplementary Figure 1H). On the contrary, cell division cycle 25C (CDC25C), CDC28 protein kinase regulatory subunit 1B (CKS1B), protein kinase, DNA-activated, catalytic subunit (PRKDC), polo like kinase 1 (PLK1), cyclin dependent kinase 1 (CDK1), and DNA topoisomerase II alpha (TOP2A), playing essential roles in the progression of cell cycle (van den Heuvel, 2005; Zhan et al., 2007; Gurley et al., 2017; Lee and Berger, 2019), were significantly down-regulated by anlotinib (Figure 2C and Supplementary Figure 1H). Western blot of phosphorylated H2AX (γH2AX), a reflection of DNA double-strand breaks (DSBs), showed a considerably up-regulation of the protein in pancreatic cancer cells treated with anlotinib (Supplementary Figure 2A). Besides, cell cycle assay revealed that the ratio of pancreatic cancer cells in G2/M phase was dramatically elevated, and that of cells in G1 phase was significantly declined, after anlotinib treatment (*p* < 0.0001, Figures 2D–G).

### Proteomics Analysis

**FIGURE 3:**
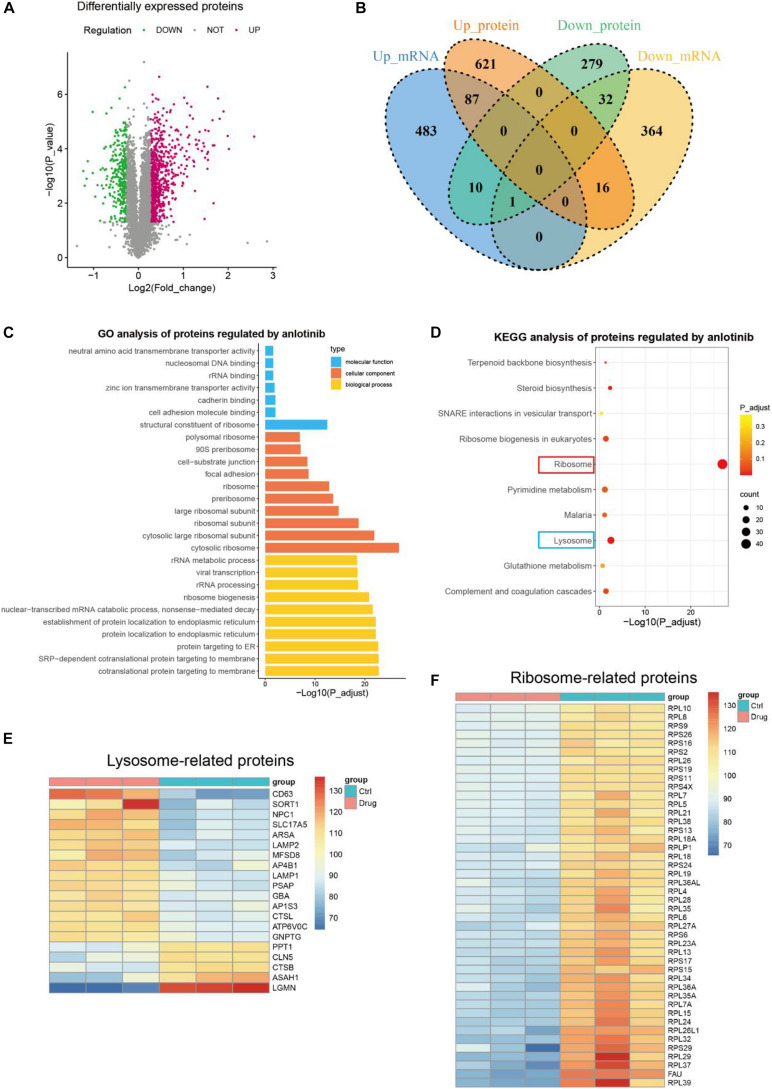
Proteomics profiling. **(A)** The volcano plot of differentially expressed proteins of PANC-1 cells treated with anlotinib. **(B)** The venn plot of DEGs and differentially expressed proteins of PANC-1 cells treated with anlotinib. **(C,D)** GO **(C)** and KEGG **(D)** analysis of differentially expressed proteins of PANC-1 cells treated with anlotinib. **(E)** The heatmap of differentially expressed proteins related to *lysosome*. **(F)** The heatmap of differentially expressed proteins related to *ribosome*.

To investigate whether the impact of anlotinib on transcriptional regulation is translated at the protein level, we further conducted proteomics analysis. A total of 67,028 unique peptides and 7,380 corresponding proteins were identified. 1,046 of them were identified as differentially expressed proteins with the following cutoff value: fold change > 1.2 and *p*-value < 0.05 (Figure 3A). Interestingly, among the 725 up-regulated proteins by anlotinib, only 87 proteins were also up-regulated at the transcriptional level, 16 proteins was even down-regulated at the mRNA level, while 621 proteins exhibited no change at the transcriptional level, suggesting most of the differentially up-regulated proteins were not affected by anlotinib at the mRNA level (Figure 3B). A similar phenomenon was also observed for the 321 down-regulated proteins (Figure 3B). This integration of transcriptomics and proteomics data inferred that a post-transcriptional regulatory mechanism was activated by anlotinib. Thus, enrichment analysis was carried out. GO analysis of these differentially expressed proteins suggested that most of them were components of ribosome and participated in rRNA binding, ribosome biogenesis, acting as structural constituent of ribosome et al. (Figure 3C). Consistently, ribosome was the most significantly enriched term as indicated by KEGG analysis (red frame, Figure 3D). In addition, we noticed that lysosome was the second significantly enriched term (blue frame, Figure 3D), and most of the lysosome-related proteins, like LAPM1 and LAMP2, were significantly up-regulated by anlotinib (15/20, Figure 3E), suggesting an increased formation of lysosome after the drug treatment. Meanwhile, all the 44 ribosome-related proteins were significantly down-regulated by anlotinib, probably through proteolysis since only 5 of these ribosome-related proteins showed a change at the mRNA level (Figure 3F and Supplementary Figure 2B).

### Integrated Proteomics and Phosphoproteomics Analysis

**FIGURE 4:**
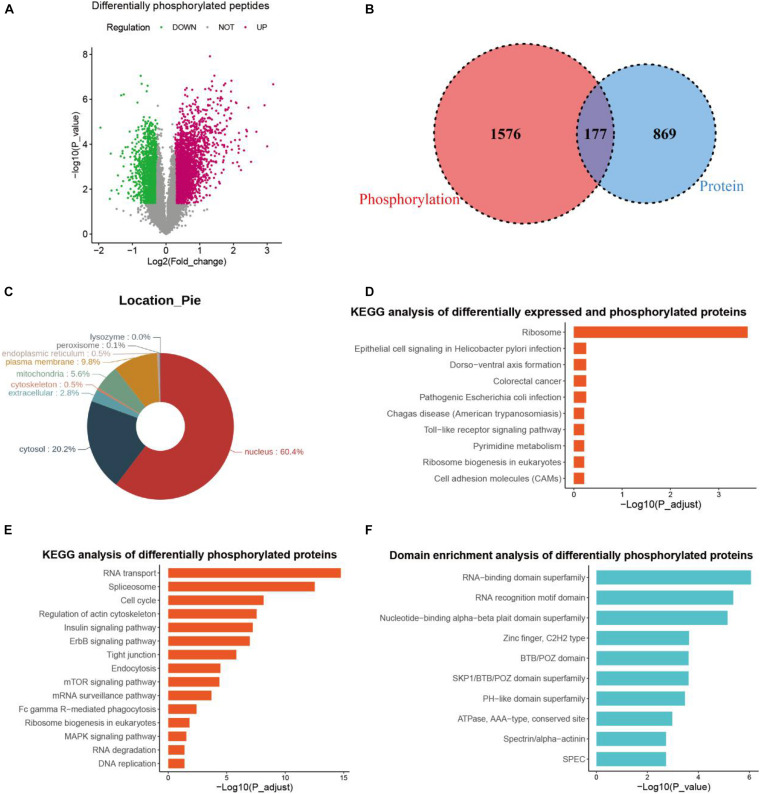
Phosphoproteomics profiling. **(A)** The volcano plot of differentially phosphorylated peptides of PANC-1 cells treated with anlotinib. **(B)** The venn plot of differentially expressed proteins and differentially phosphorylated proteins of PANC-1 cells treated with anlotinib. **(C)** Cellular location analysis of differentially phosphorylated proteins of PANC-1 cells treated with anlotinib. **(D)** KEGG analysis of differentially expressed and phosphorylated proteins of PANC-1 cells treated with anlotinib. **(E)** KEGG analysis of all differentially phosphorylated proteins of PANC-1 cells treated with anlotinib. **(F)** Domain enrichment analysis of all differentially phosphorylated proteins of PANC-1 cells treated with anlotinib.

As a TKI targeting several growth factor receptors and c-kit, anlotinib exerts impact on the phosphorylation of downstream mediators like Akt (He et al., 2018; Xie et al., 2018; Zhou A. P. et al., 2019). In addition, phosphorylation and de-phosphorylation is a major post-translation modification that regulates multiple cellular functions like cell growth and apoptosis (Singh et al., 2017). To better understand the role of anlotinib in suppressing pancreatic cancer cells, we also conducted phosphoproteomics analysis, which identified 4 323 differentially phosphorylated peptides (Figure 4A), reflecting an altered change at the phosphorylation level of 2,030 proteins. Among them, 277 proteins contained both up-regulated and down-regulated phosphorylated peptides, suggesting the regulation of phosphorylation occurred at multiple sites of these proteins. Consequently, a total of 1,753 proteins were regulated at the phosphorylation level by anlotinib. Most of these proteins located in the nucleus (60.4%), and 20.2% of them in cytosol (Figure 4B). Besides, a majority of these differentially phosphorylated proteins (1576/1753) showed no difference at the protein level after anlotinib treatment, and vice versa, most of the differentially expressed proteins (869/1046) were not phosphorylated or de-phosphorylated by the drug (Figure 4C). KEGG analysis of the 177 differentially expressed and phosphorylated proteins revealed that these proteins enriched in ribosome (Figures 4C,D). In addition, KEGG analysis of all the 1753 differentially phosphorylated proteins indicated that they were significantly enriched in RNA transport, spliceosome, cell cycle, regulation of actin cytoskeleton, mTOR signaling et al. (Figure 4E). The InterPro database^1^, an integrated documentation resource for protein families, domains, and functional sites (Mitchell et al., 2019), revealed that these proteins had a significant enrichment in RNA-binding domain superfamily, RNA recognition motif domain, and nucleotide-binding alpha-beta plait domain superfamily (Figure 4F).

### Anlotinib-Related Signature

**FIGURE 5:**
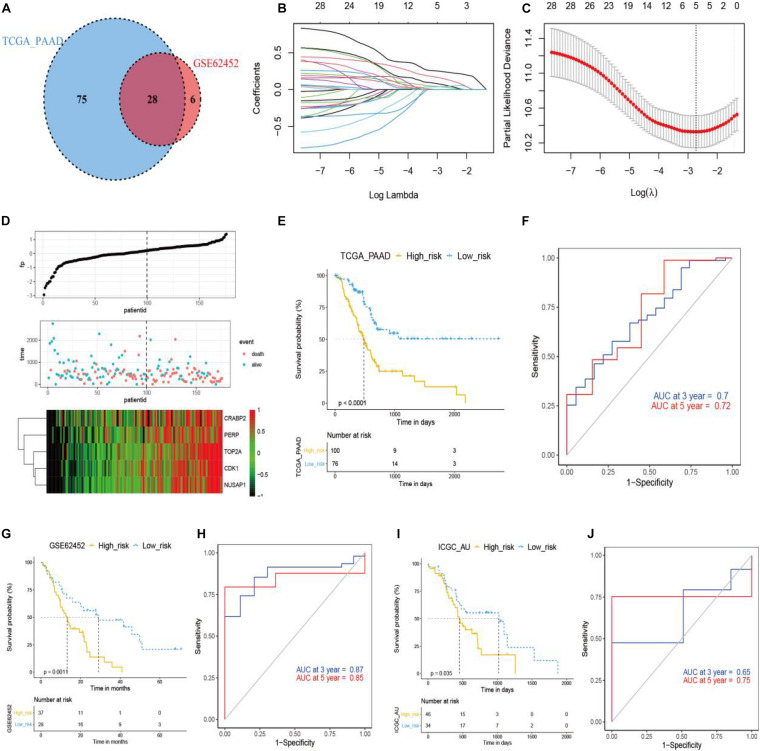
Development and validation of anlotinib-related prognostic model in pancreatic cancer. **(A)** The venn plot of anlotinib-induced DEGs with prognostic relevance in TCGA_PAAD and GSE62452 data sets. **(B,C)** LASSO Cox regression analysis of anlotinib-induced DEGs in TCGA_PAAD data set, with the tuning parameter (λ) calculated based on partial likelihood deviance with tenfold cross-validation. An optimal log λ value was shown by the vertical black line in the plot. **(D)** The distribution of risk scores, survival status and expression of five crucial genes in patients of the TCGA_PAAD data set. **(E,F)** Kaplan–Meier plots **(E)** and time-dependent ROC analysis **(F)** of the risk score regarding OS and survival status in the TCGA_PAAD cohort. **(G,H)** Kaplan–Meier plots **(G)** and time-dependent ROC analysis **(H)** of the risk score regarding OS and survival status in the GSE62452 cohort. **(I,J)** Kaplan–Meier plots **(I)** and time-dependent ROC analysis **(J)** of the risk score regarding OS and survival status in the ICGC_AU cohort.

Next, we intended to identify pancreatic cancer patients who would respond to anlotinib, based on the transcriptomics result in this work and available data deposited in public databases like TCGA. To do this, we first screened genes that were down-regulated by anlotinib at the transcriptional level and were risk factors in pancreatic cancer identified by univariate Cox analysis. 113 and 34 anlotinib-regulated genes were identified to be risk factors in the TCGA_PAAD and GSE62452 datasets, respectively (Figure 5A and Supplementary Table 3). The shared 28 risk factors in these two datasets were input into a LASSO Cox regression model, which outputted five crucial genes: TOP2A, CRABP2, CDK1, NUSAP1, and PERP (Figures 5B,C). These five genes were significantly up-regulated in pancreatic cancer when compared with corresponding normal tissues (Supplementary Figures 2C–H). Further, the risk score was calculated based on the aforementioned formula. Pancreatic cancer patients in the TCGA_PAAD dataset were stratified into a high-risk (*n* = 100) or a low-risk subgroup (*n* = 76) based on the optimal cut-off value (0.5) of the risk score (Supplementary Figure 2I). Figure 5D demonstrated the occurrences of death and expression of the five crucial genes in the high- and low-risk populations. Survival analyses inferred that pancreatic cancer patients in the high-risk subgroup had a significantly shorter survival time than those in the low-risk one (Figure 5E, *P* < 0.0001). As shown in Figure 5F, the AUC of the time-dependent ROC curves reached 0.7 at 3 year and 0.72 at 5 years, suggesting a favorable predictive value of the risk score. To validate the indicative value of the risk score in other datasets, we calculated the risk score in the GSE62452 (*n* = 65) and ICGC_AU (*n* = 80) datasets using the same risk formula and cutoff point obtained from the TCGA_PAAD data set. 57% of pancreatic cancer patients in the GSE62452 cohort (*n* = 37) and in the ICGC_AU cohort (*n* = 46) were categorized into high-risk subgroup while the rest patients were into low-risk subgroup. Consistent with the result from the TCGA_PAAD data set, patients in the high-risk subgroup of the GSE62452 and ICGC_AU cohorts exhibited a significantly shorter OS than those in the corresponding low-risk subgroup (Figures 5G,I). The AUC for OS was calculated to be 0.87 at 3 years and 0.85 at 5 years in the GSE62452 cohort (Figure 5H), and was 0.65 at 3 years and 0.75 at 5 years in the ICGC_AU cohort (Figure 5J).

**FIGURE 6:**
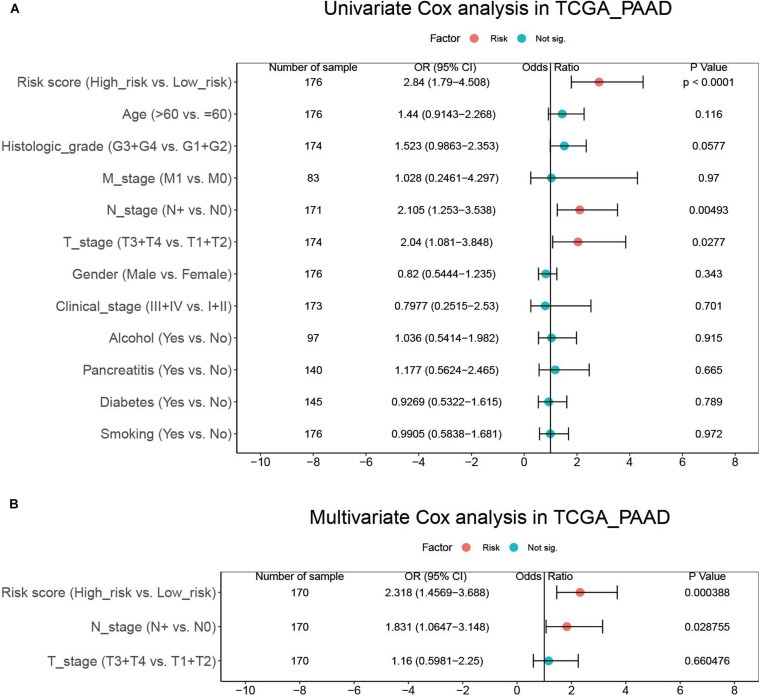
Results of the univariate **(A)** and multivariate **(B)** Cox regression analyses regarding OS in the TCGA_PAAD cohort.

To evaluate whether the risk score could serve as an independent prognostic factor, we first conducted univariate Cox analysis, which revealed that risk score, N stage, and T stage had significant prognostic relevance (Figure 6A). These three factors were then underwent multivariate Cox analysis, which demonstrated that the risk score (HR = 2.318, 95% CI = 1.4569–3.688, *p* = 0.000388) and N stage (HR = 1.831, 95% CI = 1.0647–3.148, *p* = 0.028755) were independent prognostic factors (Figure 6B).

**FIGURE 7:**
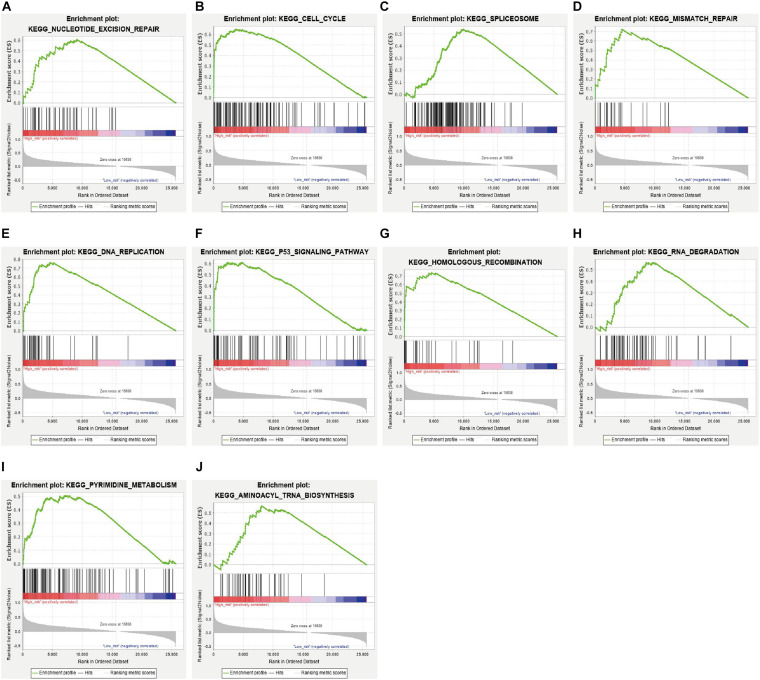
GSEA between PAAD patients with high risk score and those with low risk score **(A–J)**.

In addition, we analyzed the difference in the pathways between the high- and low-risk subgroups by conducting GSEA. A total of 33 pathways were significantly enriched in the high-risk subgroup (Supplementary Table 4). These pathways included DNA damage response pathways like nucleotide excision repair, mismatch repair, homologous recombination and base excision repair; cell cycle related pathways like cell cycle, p53 signaling pathway, DNA replication and oocyte meiosis; RNA metabolism related pathways like spliceosome, RNA degradation, aminoacyl tRNA biosynthesis; and cancers like pancreatic cancer, small cell lung cancer (Figures 7A–J and Supplementary Table 4).

## Discussion

Angiogenesis contributes to tumor growth and disease progression, and targeting this process has been proved effective in cancer treatment (Zhao and Adjei, 2015). Anlotinib, a novel TKI inhibiting multiple pro-angiogenetic signaling pathways, shows therapeutic effects against several types of cancer with good tolerance (Chi et al., 2018; Lin et al., 2018; Sun et al., 2018; Xie et al., 2018; Zhou A. P. et al., 2019). Hence, the agent might be a potential candidate for advanced pancreatic cancer, a disease with few breakthroughs in therapy and with extremely low 5-year survival rate (Kleeff et al., 2016).

Anlotinib inhibits proliferation and induces apoptosis of pancreatic cancer cells, as shown in this work and in a recent study by Yang L. et al. (2020). In addition, our work also revealed that anlotinib impairs the migration and invasion capability of pancreatic cancer cells (Figures 1E–H). Some other studies reported that anlotinib suppresses progression of osteosarcoma, hepatocellular carcinoma, synovial sarcoma, intrahepatic cholangiocarcinoma, colorectal cancer, et al. (He et al., 2018; Tang et al., 2019; Wang et al., 2019; Song et al., 2020; Yang Q. et al., 2020). Taken together, these data suggested a broad cytotoxicity of anlotinib on multiple types of tumor.

To shed light on the mechanism of anlotinib in pancreatic cancer, we first conducted transcriptomics analysis, which revealed that ER stress related pathways (such as *unfold protein response* and *NRF2-mediated oxidative stress response*), cell cycle related pathways (such as *Cell cycle: G2/M DNA damage checkpoint regulation* and *cell cycle control of chromosomal replication*) and DNA damage related pathways were significantly enriched terms (Figure 2B). Anlotinib caused DNA damage, as supported by an increased level of γH2AX and up-regulation of DNA damage induced genes like ATM and GADD45A. Several genes play extremely essential roles in repairing DNA damage and save cells from apoptosis. For instance, ATM initiates DNA damage repair (DDR) by phosphorylating a variety of targets such as BRCA1 DNA repair associated (BRCA1), which functions in the repair of DSBs by homologous recombination (Goldstein and Kastan, 2015; Zhao et al., 2017). ATM also activates p53, which plays a prominent role in DNA repair (Goldstein and Kastan, 2015; Williams and Schumacher, 2016). The canonical pathway analysis of DEGs in this work demonstrated that both *p53 signaling* and *role of BRCA1 in DNA damage response* are significantly affected pathways by the drug (Figure 2B). In addition, DNA damage arrests cell cycle progression so as to spare time for damaged cells to repair (Sancar et al., 2004). This phenomenon is observed in this work, as cell cycle assay showed that the ratio of pancreatic cancer cells in G2/M phase was dramatically elevated after anlotinib treatment (Figures 2D–G). Transcriptomics analysis has been applied to explore the mechanism of anlotinib in several tumors like intrahepatic cholangiocarcinoma, pancreatic cancer, colon cancer, and synovial sarcoma (Tang et al., 2019; Song et al., 2020; Sun et al., 2020; Yang L. et al., 2020). Yang L. et al. (2020) reported that DEGs in pancreatic cancer cells with anlotinib treatment are associated with apoptosis and ER stress, and demonstrated that anlotinib induces apoptosis of pancreatic cancer cells through the activation of ER stress via PERK/p-eIF2α/ATF4 pathway, supporting the findings in this work. KEGG enrichment analysis based on anlotinib induced DEGs in intrahepatic cholangiocarcinoma revealed that cell cycle is the most significantly enriched pathway (Song et al., 2020). Canonical pathway analysis of DEGs in synovial sarcoma with anlotinib treatment showed that *mitotic roles of Polo-Like kinas* is the most significantly activated terms (Tang et al., 2019). Taken together, transcriptomics analysis and corresponding validating experiments in this work and in other studies revealed that anlotinib has a profound impact on cell cycle progression, causing cell cycle arrest and proliferation inhibition.

Transcriptomics profiling only provides partial information about the regulatory mechanism of anlotinib. Proteomics profiling in this work revealed that most of the differentially expressed proteins are not regulated by anlotinib at the transcriptional level (Figure 3B). GO and KEGG analysis of these differentially expressed proteins showed that they are significantly enriched in ribosome and lysosome, with all ribosome-related proteins down-regulated whereas most of the lysosome-related proteins up-regulated by anlotinib (Figures 3C–F). Ribosome is required for protein synthesis and participates in protein folding (Orelle et al., 2015; Kaiser and Liu, 2018). Hyperactive ribosome biogenesis occurs in cancer and is essential to support tumor proliferation and growth (Pelletier et al., 2018; Penzo et al., 2019). Some recent studies indicated that aberrant regulation of ribosomes drives tumorigenesis and is required for the epithelial-mesenchymal transition (EMT) of cancer cells during tumor invasion (Pelletier et al., 2018; Prakash et al., 2019). CX-5461, the first-in-class selective ribosome DNA (rDNA) transcription inhibitor, exerts anti-tumor effect on advanced hematologic cancer patients (Khot et al., 2019). Thus, the destruction of ribosome-related proteins by anlotinib reflects an impairment of ribosome biogenesis in pancreatic cancer cells and might account for the inhibition of the proliferative and invasive phenotype of these cells. The activated lysosome in pancreatic cancer cells with anlotinib treatment might contribute to the destruction of ribosome-related proteins, which undergo post-transcriptional regulation as suggested by integrated analyses of transcriptomic and proteomic profiling in this study (Figure 3F and Supplementary Figure 2B). Sun et al. (2020) reported that anlotinib induces lysosomal biogenesis and activates lysosomal function in colon cancer cells, suggesting the positive impact of anlotinib on lysosome biogenesis might be shared in other types of tumor.

As a TKI, anlotinib inhibits phosphorylation of some downstream mediators like Akt (He et al., 2018; Xie et al., 2018; Zhou A. P. et al., 2019). However, a comprehensive understanding of the impact of anlotinib on the phosphorylation of proteins in cancer cells is still lacking. In pancreatic cancer cells, only a small fraction of the differentially phosphorylated proteins (177/1593) is also affected at the protein level by anlotinib (Figure 4B), suggesting different mechanisms other than ribosome and lysosome might be activated by anlotinib at the phosphorylation level. KEGG analysis of the aforementioned 177 proteins revealed that they are enriched in ribosome (Figure 4D), suggesting ribosome-related proteins are not only down-regulated at the protein level, but are differentially phosphorylated. Modifications in the intrinsic components of ribosome, such as phosphorylation and ubiquitylation, are gradually recognized as important mechanisms for translational control, and are intimately linked to human disease (Simsek and Barna, 2017). However, our understanding about the impact of these modifications in ribosome-related proteins is limited, thus how anlotinib regulates mRNA translation through phosphorylation of these ribosome-related proteins requires further investigation. Anlotinib can inhibit the phosphorylation of Akt and mTOR in several types of cancer like hepatocellular carcinoma, colon cancer and intrahepatic cholangiocarcinoma (He et al., 2018; Song et al., 2020; Sun et al., 2020; Yang Q. et al., 2020), supporting the phosphoproteomics analysis in this work (Figure 4F). Cell cycle and DNA replication is predicted to be inhibited by anlotinib via regulation on the phosphorylation of cell cycle related proteins. For instance, hyperphosphorylated Cdc25C is essential and required in inducing G2/M phase transition by dephosphorylating Cdc2/cyclin B-Cdk1 (Wang et al., 2007; Xia et al., 2019). However, Cdc25C is significantly down-phosphorylated whereas Cdk1 up-phosphorylated by anlotinib (Figure 4F and Supplementary Figure 2J). Interestingly, domain enrichment analysis of these 1593 differentially phosphorylated proteins suggested that the mostly affected terms include RNA-binding domain superfamily and RNA recognition motif domain, and the most significantly enriched term of these differentially phosphorylated proteins is RNA transport. In addition, other RNA processing related pathways, like spliceosome, mRNA surveillance pathway, and RNA degradation were also significantly enriched terms (Figure 4F). These data suggested that a disruption in RNA processing might occur in pancreatic cancer cells after anlotinib treatment.

Based on the transcriptomics profiling of anlotinib in this work and available pancreatic cancer related data deposited in TCGA, GEO, and ICGC databases, we further constructed a prognostic model which consists of five crucial genes, namely TOP2A, CRABP2, CDK1, NUSAP1, and PERP. All these five genes were significantly up-regulated in pancreatic cancer (Supplementary Figures 2C–H). TOP2A and CDK1 are cell cycle-related genes and they facilitate proliferation and invasion of pancreatic cancer (Feng et al., 2015; Pei et al., 2018; Piao et al., 2019). The role of CRABP2 and NUSAP1 in pancreatic cancer is currently unclear, but CRABP2 and NUSAP1 favors proliferation and invasion of several types of tumor, such as prostate, lung, gastric and colorectal cancer (Gordon et al., 2017; Han G. et al., 2018; Wu et al., 2019; Ge et al., 2020; Xu et al., 2020). Interestingly, a recent study showed that knocking down the expression of PERP in pancreatic cancer cells promotes proliferation and invasion of the cells, suggesting a tumor-suppressive function of PERP in pancreatic cancer (Wang et al., 2020). Indeed, PERP has a well-known pro-apoptotic function by dependently or independently of p53 signal pathways, and loss of the gene is linked to tumorigenesis (Attardi et al., 2000; Beaudry et al., 2010; McDonnell et al., 2019; Awais et al., 2016). Pancreatic cancer has a significantly elevated expression of PERP, and PERP serves as a risk factor based on univariate Cox analysis (Supplementary Table 2), suggesting an oncogenic role of PERP in pancreatic cancer. A possible explanation to this contradiction is that the increased transcriptional level of PERP might be a feedback to a decreased protein level of PERP or to an inhibition of the downstream effector of PERP in this cancer, but future studies are required to solve this problem. The prognostic gene signature constructed in this work has a good predictability in the training and validating datasets (Figures 5F,G,H). Further, the high-risk subgroup, stratified by the gene signature, has a significant enrichment in several signaling pathways that are regulated by anlotinib, such as cell cycle, spliceosome, DNA replication, RNA degradation, DNA damage repair and p53 signaling pathway (Figures 2B, 4F, 7A–J), suggesting the subgroup might have a good response to the drug.

Besides, this work has several limitations that should be pointed out. Firstly, this work aimed to provide a general view of the impact of anlotinib on the transcriptional, protein and phosphorylation regulation in pancreatic cancer, and specific signaling pathways were not analyzed in details. Second, the validity of the gene signature in predicting response to anlotinib should be tested by well-designed prospective clinical trials. Thirdly, a predictive model based on IHC staining on tissue slides is more convenient; however, most differentially expressed proteins in pancreatic cancer cells after anlotinib treatment had no change on the transcriptional level, thus a novel model, based on IHC staining of ribosme-related proteins, might be more applicable for clinical practice.

## Conclusion

In conclusion, anlotinib exerts cytotoxic effects on pancreatic cancer through different mechanisms at the transcriptional, protein and phosphorylation level. Based on the transcriptomics profiling analysis of anlotinib in pancreatic cancer and RNA-seq data in public datasets, a novel gene signature is developed to predict the prognosis of pancreatic cancer patients and to help selecting patients who might be responsive to anlotinib.

## Data Availability Statement

The datasets presented in this study can be found in online repositories. The names of the repository/repositories and accession number(s) can be found in the article/Supplementary Material.

## Author Contributions

XZ and YL performed the *in vitro* experiments and collection of data. XZ, YL, and ZS carried out the analysis of data. ZS, ZZ, and HO conducted the bioinformatics analysis. JL, JT, and JZ gave useful discussion and advice. ZS drafted the manuscript. All authors revised the manuscript before the final version was approved to be published.

## Conflict of Interest

The authors declare that the research was conducted in the absence of any commercial or financial relationships that could be construed as a potential conflict of interest.

## Footnotes

1 http://www.ebi.ac.uk/interpro/
