# Supplementary material for: Multi-Omics Analysis of Anlotinib in Pancreatic Cancer and Development of an Anlotinib-Related Prognostic Signature
Source: Front Cell Dev Biol. 2021 Mar 4;9:649265. doi: 10.3389/fcell.2021.649265 (PMC7969999; doi:10.3389/fcell.2021.649265)

Supplementary Figure legends

Supplementary Figure 1. (A-B) Determination of IC50 values of anlotinib in PANC-1 (A) and AsPC-1 (B) cells. (C) The distribution of the strength of signals shown by relative signal box plot. (D) The intra-class and inter-class degree of variance as reflected by principal component analysis. (E) The correlation of the strength of signals between samples as shown by correlation analysis. (F-G) RT-PCR of 15 randomly selected down-regulated genes (F) and 15 up-regulated genes (G) induced by anlotinib. (H) RT-PCR of CDC25C, CKS1B, PRKDC, PLK1, CDK1, TOP2A, ATM, SFN and GADD45A.

Supplementary Figure 2. (A) Western blot of phosphorylated H2AX (γH2AX) in PANC-1 and AsPC-1 cells treated with anlotinib or DMSO. (B) Differentially expressed ribosome-related genes at transcriptional level in PANC-1 cells induced by anlotinib. (C) Expression of 5 crucial genes in pancreatic tumor tissues when compared with normal samples in GSE62165. (D-H) Expression of 5 crucial genes in pancreatic tumor tissues when compared with normal samples based on the GEPIA2 database. (I) Determination of the best cutoff value of the risk score. (J) CDC25C is down-phosphorylated and CDK1 up-phosphorylated by anlotinib in pancreatic cancer cells.

Supplementary Figure 1


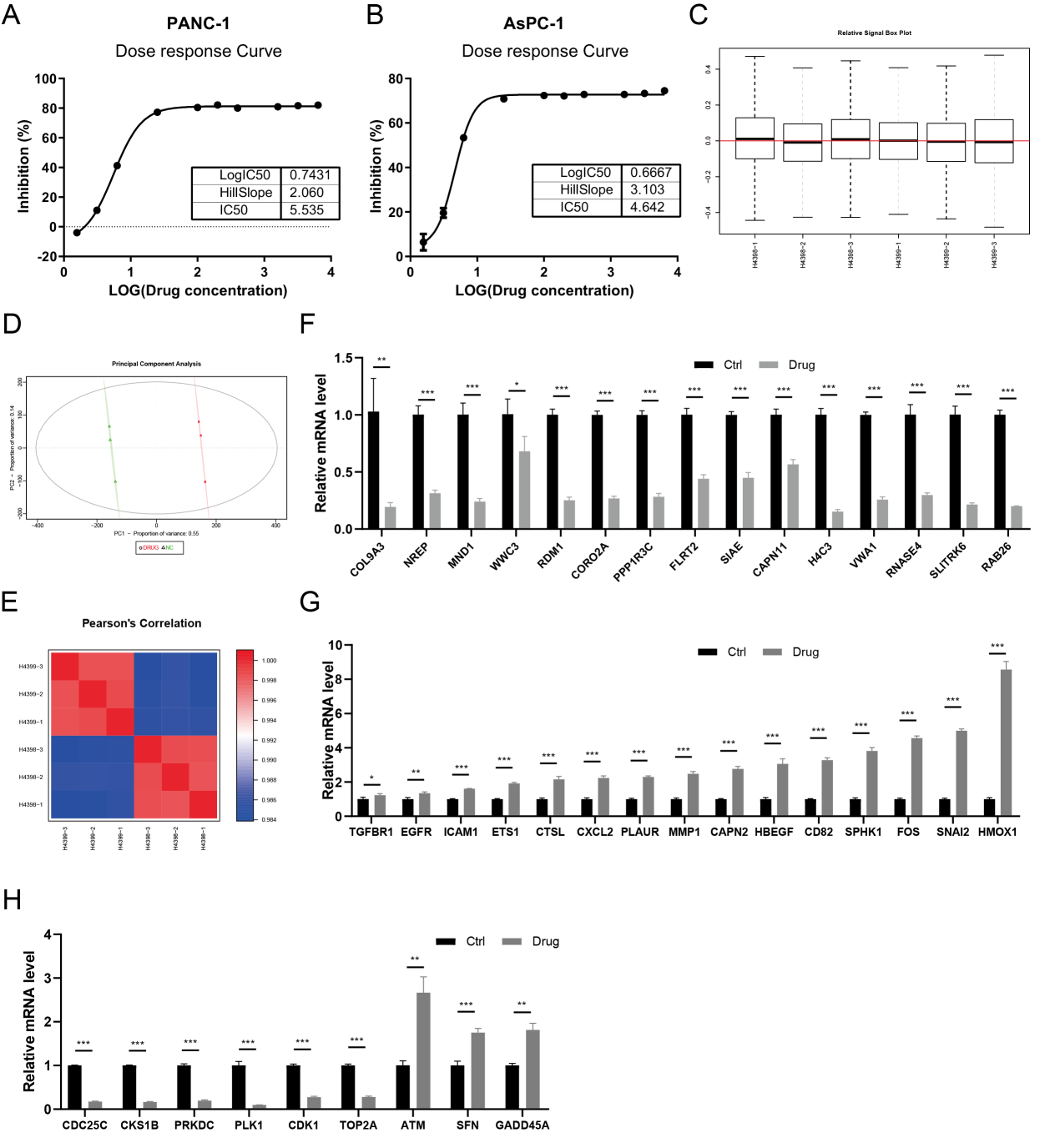


Supplementary Figure 2


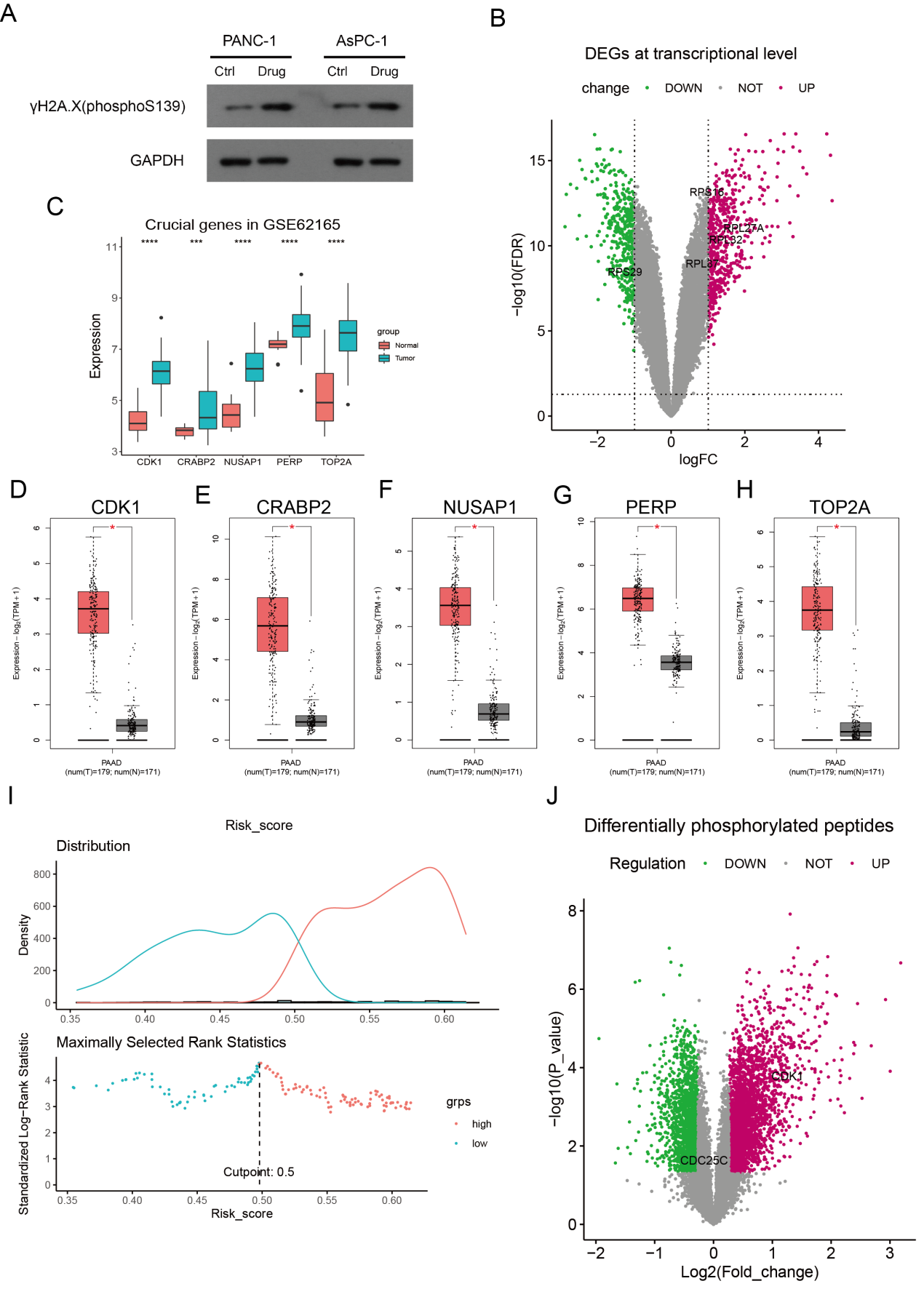

Supplement: Supplementary file 1 [file Data_Sheet_1.docx]
